# Genes Expressed Differentially in Hessian Fly Larvae Feeding in Resistant and Susceptible Plants

**DOI:** 10.3390/ijms17081324

**Published:** 2016-08-12

**Authors:** Ming-Shun Chen, Sanzhen Liu, Haiyan Wang, Xiaoyan Cheng, Mustapha El Bouhssini, R. Jeff Whitworth

**Affiliations:** 1 Hard Winter Wheat Genetics Research Unit, USDA-ARS, 4008 Throckmorton, Kansas State University, Manhattan, KS 66506, USA; 2 Department of Entomology, Kansas State University, Manhattan, KS 66506, USA; xycheng@ksu.edu (X.C.); jwhitwor@k-state.edu (R.J.W.); 3 Department of Plant Pathology, Kansas State University, Manhattan, KS 66506, USA; liu3zhen@ksu.edu; 4 Department of Statistics, Kansas State University, Manhattan, KS 66506, USA; hwang@ksu.edu; 5 International Center for Agricultural Research in the Dry Area, Rabat 10106, Morocco; M.BOHSSINI@cgiar.org

**Keywords:** plant resistance, effectors, compatible interaction, incompatible interaction, salivary glands, cytochrome P450, RNA-sequencing, gall midge

## Abstract

The Hessian fly, *Mayetiola destructor*, is a destructive pest of wheat worldwide and mainly controlled by deploying resistant cultivars. In this study, we investigated the genes that were expressed differentially between larvae in resistant plants and those in susceptible plants through RNA sequencing on the Illumina platform. Informative genes were 11,832, 14,861, 15,708, and 15,071 for the comparisons between larvae in resistant versus susceptible plants for 0.5, 1, 3, and 5 days, respectively, after larvae had reached the feeding site. The transcript abundance corresponding to 5401, 6902, 8457, and 5202 of the informative genes exhibited significant differences (*p* ≤ 0.05) in the respective paired comparisons. Overall, genes involved in nutrient metabolism, RNA and protein synthesis exhibited lower transcript abundance in larvae from resistant plants, indicating that resistant plants inhibited nutrient metabolism and protein production in larvae. Interestingly, the numbers of cytochrome P450 genes with higher transcript abundance in larvae from resistant plants were comparable to, or higher than those with lower transcript abundance, indicating that toxic chemicals from resistant plants may have played important roles in Hessian fly larval death. Our study also identified several families of genes encoding secreted salivary gland proteins (SSGPs) that were expressed at early stage of 1^st^ instar larvae and with more genes with higher transcript abundance in larvae from resistant plants. Those SSGPs are candidate effectors with important roles in plant manipulation.

## 1. Introduction

The Hessian fly, *Mayetiola destructor*, is a parasitic pest of wheat plants and causes serious yield loss in nearly all wheat regions in USA, North Africa, and Europe [1,2,3]. Like biotrophic plant pathogens, the Hessian fly interacts with wheat in a typical gene-for-gene relationship, namely, for every resistance (R) gene in the host plant, there is a corresponding avirulence (Avr) gene in the pathogen or insect parasite [4,5]. So far, 34 R genes have been identified and are named *H1* to *H32*, *Hdic*, and *H34* [6]. All 34 R genes are dominant except *h4*, which is recessive, and all are single effective genes except for *H7H8*, which need to be together to be effective. No Hessian fly R gene in wheat has been cloned and characterized. However, several lines of evidence suggest that Hessian fly R genes share structural similarity with plant disease R genes and belong to the super-group of receptor-like kinases which possess a nucleotide binding site and leucine zip repeats. Two Hessian fly Avr genes have been cloned and characterized [7,8]. These Avr genes encode secreted proteins that are likely injected into plant tissue through saliva during feeding. Except for the presence of a typical secretion signal peptide, the structures of the two identified Avr proteins are completely different, and therefore, are likely to perform different functions.

In order to live and develop in a host plant, Hessian fly larvae need to manipulate the host plant extensively, including inhibiting plant growth, inducing the formation of nutritive cells, and preventing secondary infestation by microbes that may kill the plant [9,10]. The exact molecular mechanisms for Hessian fly larvae to manipulate host plants is not known. However, several lines of evidence suggest that Hessian fly larvae manipulate plants through the secretion of saliva that contains effector proteins. Through analyses of dissected salivary glands and genome sequencing, nearly two thousand genes were identified encoding secreted salivary gland proteins (SSGPs). The large number of SSGP-encoding genes can be classified into super-families and families based on their evolutionary relationship. A few families of SSGP genes are very big with more than 500 members. Evidence also suggests that SSGP genes are frequently duplicating and diversifying. Members from the same super-family or family share a so called unconventional conservation pattern, in which the 5′- and 3′-untranslated regions, the region encoding the secretion signal peptide, and introns are highly conserved whereas the regions encoding mature proteins are highly diversified [11,12]. This type of unconventional conservation is likely formed under high selection pressure that selects mutated SSGPs, which may allow Hessian fly to overcome plant resistance. Indeed, plant resistance to Hessian fly conferred by individual R genes is short-lived, lasting for only 6–8 years after an R gene is deployed to the field [13]. Even though numerous SSGP genes have been identified, the specific function and importance of individual SSGP genes that allows Hessian fly to parasitize wheat plants are not yet known. To identify potential key SSGP genes for further research, we hypothesize that the expression of those putative effector genes critical for Hessian fly to manipulate host plants should be elevated when Hessian fly larvae feed in resistant plants. Therefore, the first objective of this study is to examine SSGP genes expressed differentially between Hessian fly larvae feeding in resistant plants and those feeding in susceptible plants via RNAseq, and to identify potentially critical effector genes for further genetic research.

Hessian fly is mainly controlled by developing and deploying resistant wheat cultivars. Resistance in wheat to Hessian fly is by antibiosis, namely, Hessian fly larvae become physically inactive in resistant wheat after 4–5 days and eventually die before developing into second instars [3,5]. The exact mechanisms that cause Hessian fly larvae to die in resistant plants is not yet known. Different types of defensive, toxic chemicals such as protease inhibitors, reactive oxygen species, toxic lectins, and secondary metabolites were induced specifically in resistant plants upon Hessian fly larval attack [14,15,16,17,18,19,20]. Therefore, Hessian fly larvae may die due to the toxicity of the defense chemicals from host plants. On the other hand, larvae from resistant plants can survive once they are transferred onto susceptible plants [19]. The cell wall is promptly strengthened in resistant plants in response to Hessian fly larval attack, and no nutritive cells are formed at the feeding site in these plants [10,19]. These observations indicate that the death of Hessian fly larvae in resistant plants may be due to the lack of nutrients. We hypothesize that changes in gene expression in larval feeding in resistant plants reflect differences in insect physiology, and identification of those genes will reveal the mechanism for Hessian fly larval death. Therefore, the second objective of this study is to identify changes in gene expression and pathways that could help to infer molecular mechanisms for Hessian fly larval death in resistant plants.

## 2. Results

Samples from 0.5-, 1-, 3-, and 5-day larvae feeding in either resistant (wheat cultivar “Molly”) or susceptible (wheat cultivar “Newton”) plants were subjected to mRNA sequencing (RNA-Seq) using the Illumina HiSeq2000 sequencing platform (Illumina, San Diego, CA, USA). Three biological replicates for each time point were conducted. On average 33.5 million of 2 × 101 bp paired-end raw reads per sample were obtained. The raw reads were processed as described previously [21], and were mapped to the Hessian fly draft genome sequence (Mdes20100623, https://i5k.nal.usda.gov/Mayetiola_destructor) [22] via GSNAP, an intron-aware aligner [23]. A total of 18,593 informative gene models (defined in the Methods and referred to as genes hereafter) were identified.

### 2.1. Changes in Overall Gene Expression

**Figure 1:**
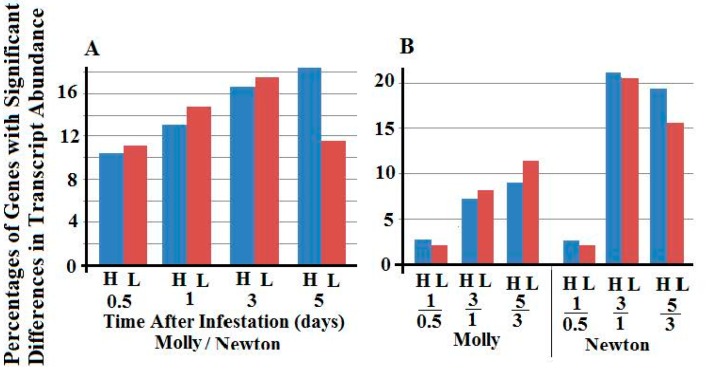
Percentages of total genes with higher (blue bars, H) and lower (red bars, L) transcript abundance. (**A**) Percentages of genes with higher and lower transcript abundance between larvae feeding in resistant (Molly) and susceptible (Newton) plants at 0.5, 1, 3, and 5 days; (**B**) Percentages of genes with higher and lower transcript abundance between two successive stages of Hessian fly larvae feeding in either resistant (Molly) or susceptible (Newton) plants.

Transcript abundance of informative genes was compared pair-wise between samples from larvae in resistant plants and those in susceptible plants at the same time. At 0.5, 1, 3, and 5 days, 11,832, 14,861, 15,708, and 15,071 genes were informative for the comparisons between larvae feeding in resistant versus susceptible plants, respectively. The transcript abundance corresponding to 5401, 6902, 8457, and 5202 of the informative genes exhibited significant differences between these paired samples at 5% false discovery rate (FDR). Further analysis revealed that the percentages of genes with higher transcript abundance in larvae feeding in resistant plants were slightly lower than the percentages of genes with lower transcript abundance, except for the sample from 5-day larvae, in which a much greater percentage of genes exhibited higher transcript in the larval sample from resistant plants (Figure 1).

In addition, the transcript abundance of the informative genes was also compared pair-wise between two successive insect stages fed on the same host, namely, samples from 1- versus 0.5-day larvae, 3- versus 1-day larvae, and 5- versus 3-day larvae fed in either resistant or susceptible plants, respectively (Figure 1B). The numbers of informative genes were 11,138, 15,530, and 16,532 for the comparisons between 1- versus 0.5-day, 3- versus 1-day, and 5- versus 3-day larvae feeding in resistant plants; and 10,957, 15,635, and 14,886 for the comparisons between 1- versus 0.5-day, 3- versus 1-day, and 5- versus 3-day larvae feeding in susceptible plants. Transcript abundance corresponding to 1143, 3640, and 4038 of the informative genes exhibited significant differences (5% FDR) between samples of 1- versus 0.5-day, 3- versus 1-day, and 5- versus 3-day of larvae feeding in resistant plants. Transcript abundance corresponding to 821, 7254, and 6080 of the informative genes exhibited significant differences (5% FDR) between samples of 1- versus 0.5-day, 3- versus 1-day, and 5- versus 3-day larvae feeding in susceptible plants, respectively. For the larvae feeding in resistant plants, the percentages of genes with significant changes in expression increased gradually as larvae advanced into later stages. Larvae feeding in susceptible plants, however, had percentages of genes with significant changes in gene expression increased dramatically when larvae advanced from 1- to 3-days.

### 2.2. Changes in Gene Expression in Specific Functional Categories

To determine what types of genes were up- or down-regulated between samples, the 18,593 informative genes were annotated based on BLASTX search results as described previously [21], and were updated when this paper was prepared. Based on updated gene annotation, 10,477 (56.3%) of the informative genes had matches with Genbank sequences with *E*-values ≤ 1 × 10^−30^. Among the matched genes, 9760 (53.5% of the total informative genes) had known functions, whereas the remaining (3.9%) had unknown functions (Table S1). Genes with known functions were divided into eight functional categories based on their Gene Ontology (GO) terms (Table S1): “nutrient metabolism” (1109, 11.4%), “reduction/oxidation (redox) and detoxification” (129, 1.3%), “structure and adhesion” (579, 5.9%), “RNA metabolism” (551, 5.6%), “protein metabolism” (1247, 12.8%), “transport” (1140, 11.7%), “regulatory proteins” (3320, 34.0%), and “SSGPs” (1685, 17.3%). Each category was further divided into subcategories (see below).

**Figure 2:**
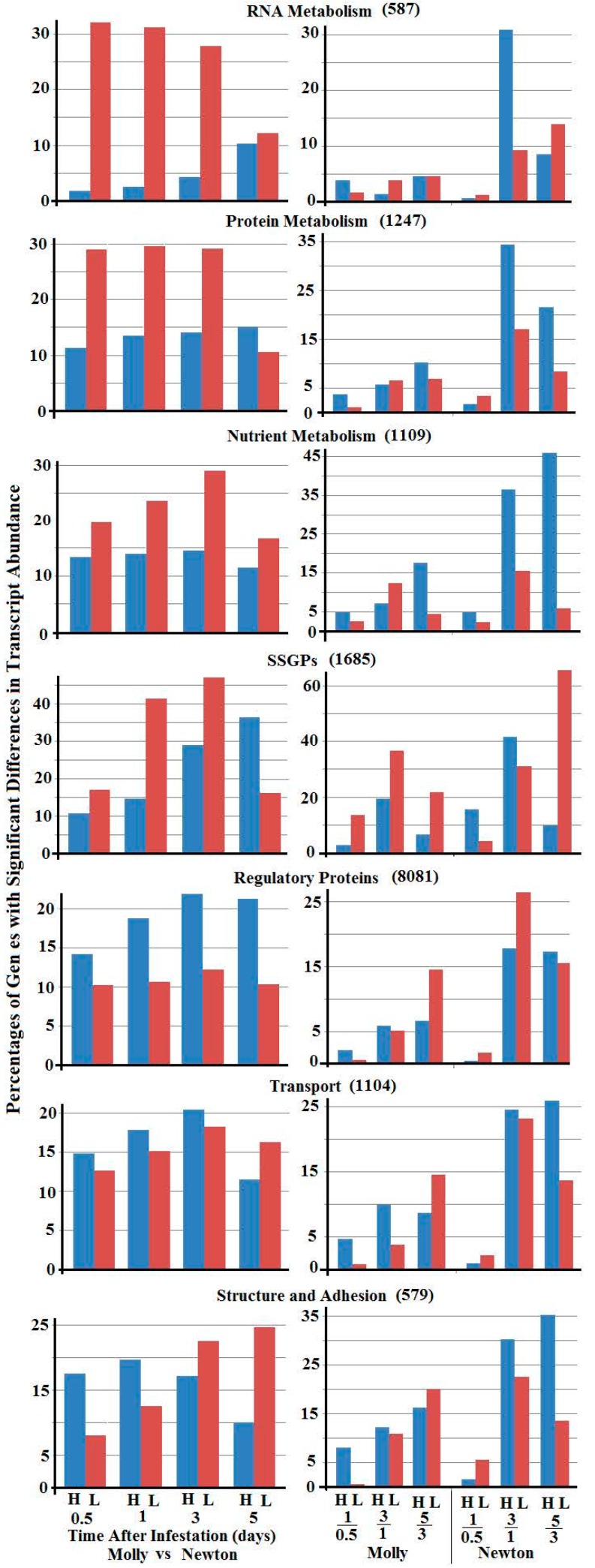
Percentages of genes in different functional categories with higher (blue bars. H) and lower (red bars, L) transcript abundance between larvae feeding in resistant (Molly) and susceptible (Newton) plants at 0.5, 1, 3, and 5 days (**left** panels), and between two successive stages (1 versus 0.5 day, 3 versus 1 day, and 5 versus 3 days) of Hessian fly larvae feeding in either resistant (Molly) or susceptible (Newton) plants (**right** panels). Gene categories are marked on each pair of graphs. The numbers of genes in each category were given in parenthesis.

Changes in gene expression in specific functional categories are shown in Figure 2, Tables S2, and S3. Genes in each functional category exhibit a different pattern (percentages) with greater or lesser transcript abundance between samples of larvae from resistant versus susceptible plants (Figure 2, left panels). The category with the most differences between larvae feeding in resistant versus susceptible plants is “RNA metabolism”. This category had over 30% of the genes exhibiting lower transcript abundance in the sample from 0.5-, 1-, and 3-day larvae from resistant plants, compared to the corresponding samples from susceptible plants, whereas less than 5% of the genes exhibited higher transcript abundance in the corresponding samples from resistant plants. Interestingly, the percentages of genes with either greater or lesser transcript abundance in 5-day larvae from resistant versus susceptible plants were similar. Three other categories including “protein metabolism”, “nutrient metabolism”, and “SSGPs” had much greater percentages of genes that exhibited lesser transcript abundance in 0.5-, 1-, and 3-day larval samples from resistant plants.

An opposite pattern was observed in the category “regulatory proteins”, in which greater percentages of genes exhibited higher transcript abundance in all samples from resistant plants compared to corresponding samples from susceptible plants. The category ‘transport’ also exhibited greater percentages of genes with greater transcript abundance in all samples except for the 5-day samples from resistant versus susceptible plants, even though the differences between greater and lesser percentages were smaller. In the category “structure and adhesion”, there were also greater percentages of genes which exhibited greater transcript abundance in the 0.5- and 1-day larval samples from resistant plants than those from susceptible plants. However, the situation was reversed and the opposite was observed in the samples from 3- and 5-day larvae.

The gene patterns with differential expression seemed to be the results of varied expression dynamics between larvae feeding in resistant versus susceptible plants. As shown in the right panels in Figure 2, the dynamics of gene expression were distinct from category to category between samples from two successive larval stages feeding in either resistant or susceptible plants.

### 2.3. Changes in Gene Expression in Specific Functional Subcategories and Pathways

**Figure 3:**
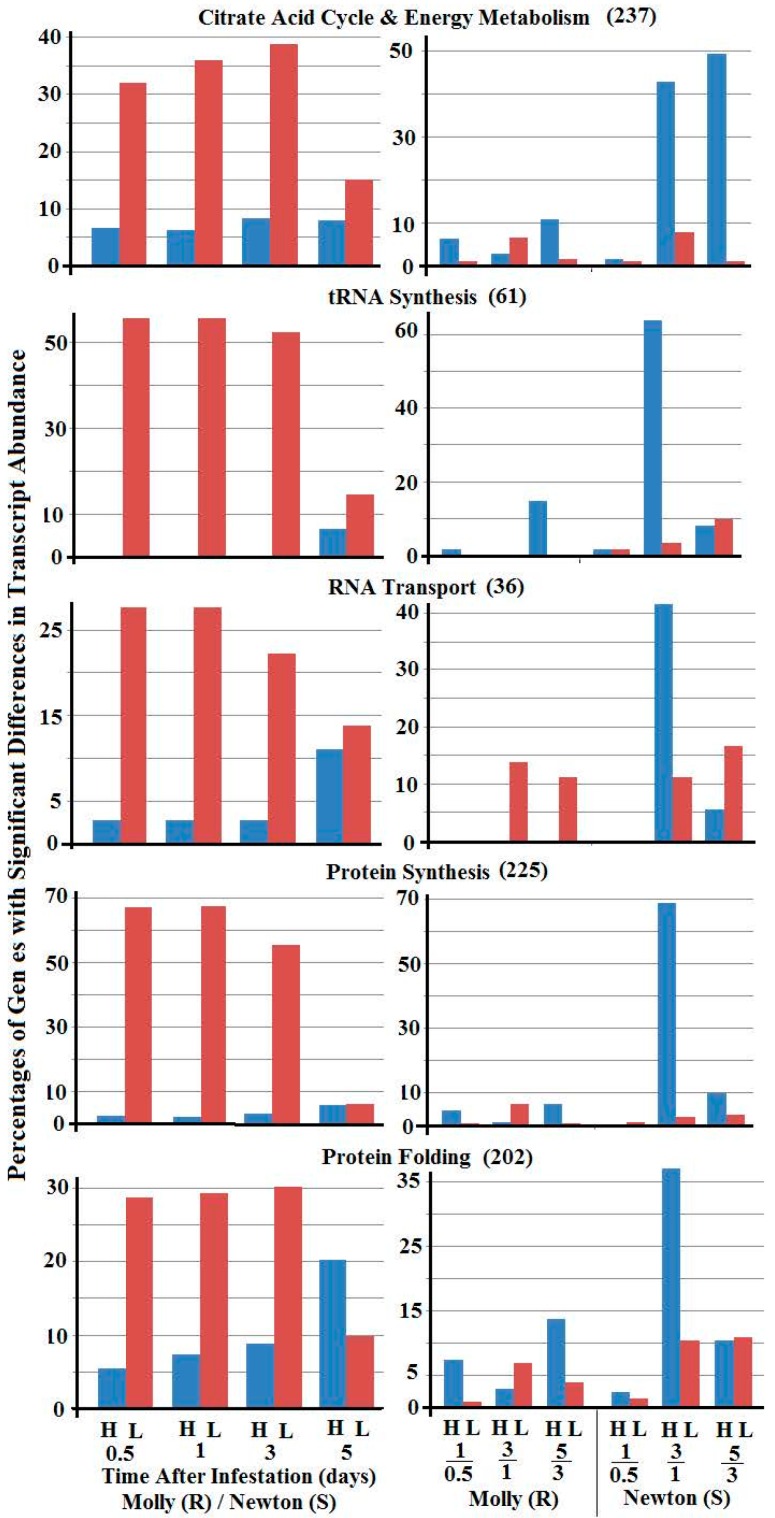
Percentages of genes in selected subcategories with higher (blue bars, H) and lower (red bars, L) transcript abundance between larvae feeding in resistant (Molly) and susceptible (Newton) plants at 0.5, 1, 3, and 5 days (**left** panels), and between two successive stages (1 versus 0.5 day, 3 versus 1 day, and 5 versus 3 days) of Hessian fly larvae feeding in either resistant (Molly) or susceptible (Newton) plants (**right** panels). Gene subcategories are marked on each pair of graphs. The numbers of genes in each subcategory were given in parenthesis.

Genes in each functional category were further divided into subcategories (Figure S1). The five subcategories with the most differences between the percentages of genes with greater and lesser transcript abundance are shown in Figure 3. These subcategories of genes are involved in “citric acid cycle & energy metabolism”, “tRNA synthesis”, “RNA transport”, “protein synthesis”, and “protein folding”. All five subcategories exhibited greater percentages of genes with lesser transcript abundance in larval samples from resistant plants than larval samples from susceptible plants.

### 2.4. Changes in Transcript Abundance of Cytochrome P450 Genes

**Figure 4:**
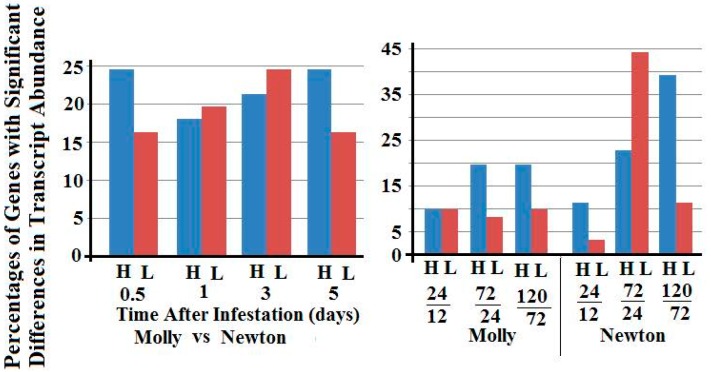
Percentages of 61 cytochrome P450 genes with higher (blue bars, H) and lower (red bars, L) transcript abundance between larvae feeding in resistant (Molly) and susceptible (Newton) plants at 0.5, 1, 3, and 5 days (**left** panels), and between two successive stages (1 versus 0.5 day, 3 versus 1 day, and 5 versus 3 days) of Hessian fly larvae feeding in either resistant (Molly) or susceptible (Newton) plants (**right** panels).

Cytochrome P450s (P450s) are one of the largest superfamilies of genes involved in a wide range of functions [24,25]. Many insect P450s are involved in detoxification of toxic chemicals ingested from host plants or produced endogenously [26,27,28]. Sixty-one P450 genes have been identified from the Hessian fly genome. Differences in transcript abundance of these genes between larvae feeding in resistant plants and those feeding in susceptible plants are shown in Figure 4A. The pattern of transcript abundance was strikingly different from that observed previously in Figure 3. For P450 genes, there were 8%–10% more genes with greater transcript abundance in 0.5- and 5-day larvae from resistant plants compared with the corresponding samples from susceptible plants. Figure 4B shows 8%–10% of P450 genes with greater transcript abundance between 3- versus 1-day and 5- versus 3-day larvae from resistant plants, indicating that the expression of P450 genes increased as larvae survived in resistant plants.

### 2.5. Changes in Transcript Abundance of Secreted Salivary Gland Proteins (SSGPs) Genes

**Figure 5:**
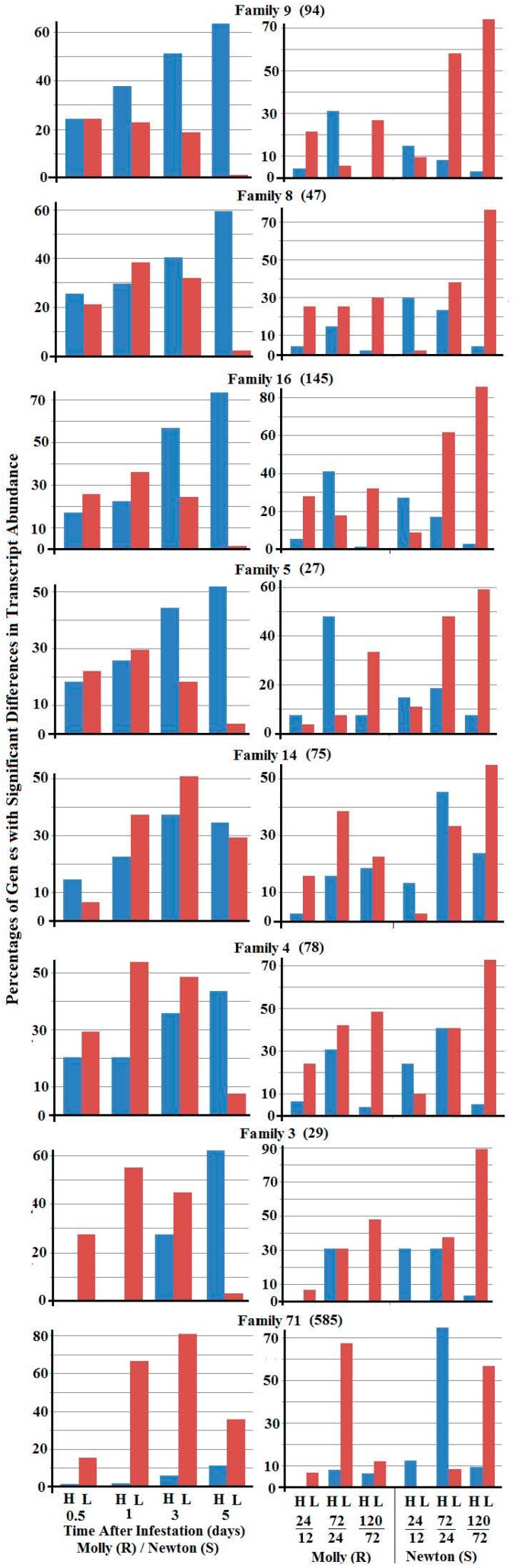
Percentages of secreted salivary gland protein (SSGP) genes in selected families with higher (blue bars, H) and lower (red bars, L) transcript abundance between larvae feeding on resistant (Molly) and two susceptible (Newton) plants at 0.5, 1, 3, and 5 days (**left** panels), and between two successive stages (1 versus 0.5 day, 3 versus 1 day, and 5 versus 3 days) of Hessian fly larvae feeding in either resistant (Molly) or susceptible (Newton) plants (**right** panels). Gene families are marked on each pair of graphs. The numbers of genes in each gene family were given in parenthesis.

The left panel of Figure 5 shows the percentages of selected SSGP gene families with greater or lesser transcript abundance between larvae from resistant plants versus those from susceptible plants. Each SSGP gene family exhibited a different pattern. For family 9, significantly more genes showed higher transcript abundance in 1-, 3-, and 5-day larvae from resistant plants versus larvae from susceptible plants. For families 8, 16, and 5, more genes exhibited greater transcript abundance in 3- and 5-day larvae from resistant plants. For families 14 and 4, more genes exhibited lower transcript abundance in 1- and 3-day larvae from resistant plants. For family 3, more genes exhibited lower transcript abundance in 0.5-, 1-, and 3-day larvae from resistant plants. For family 71, much higher percentages of genes exhibited lower transcript abundance in all stage larvae from resistant plants. The right panel of Figure 5 shows the percentages of SSGP genes with greater or lesser transcript abundance when two successive larval stages from the same type of host plants were compared. Again the pattern was distinct from gene family to gene family.

## 3. Discussion

During the long course of co-evolution, insects and host plants have formed intimate relationships, particularly for parasitic insect species. Insect attacks on plants cause extensive changes in gene expression in host plants. Identification of the changes in gene expression in host plants after an insect attack can reveal plant defense mechanisms in response to insect infestation [19]. Extensive studies have been carried out to examine differential gene expression between resistant and susceptible plants upon insect attacks through various high throughput technologies such as microarrays and RNAseq [16,29,30]. In turn, plant defensive reactions to insects may cause significant changes in gene expression in insects, and therefore, identification of the insect changes in gene expression may reveal their adaptation strategies to plant defense and death mechanisms on resistant plants. So far, very little is known about genome-wide changes in gene expression between insects feeding on resistant and susceptible plants [31].

The wheat—Hessian fly interaction results in extreme outcomes for either the infested plant or the attacking insect. During a compatible interaction, plant physiology is manipulated extensively by a Hessian fly larva, including complete inhibition of wheat growth, induction of nutritive cell formation at the feeding site, and eventual death of the plant [9]. Biochemically, growth-oriented metabolism in a susceptible plant is engineered to nutrient-accumulation-oriented metabolism by the Hessian fly, resulting in extensive pathway changes in the plant [32]. An insect feeding in a susceptible plant, however, grows normally and changes in gene expression proceed according to a development regulatory regime. During an incompatible interaction, plants resume normal growth after some initial growth deficit following the Hessian fly attack [33]. Biochemically, nutrient and energy metabolism is temporarily suppressed, whereas various plant defense pathways are activated, including the strengthening of cell walls and elevation of toxic chemicals such as reactive oxygen species, protease inhibitors, lectins, and secondary metabolites [14,15,16,17,18,19,20]. On the other hand, insects feeding in a resistant plant become inactive in 4–5 days and eventually die without developing into the second instar. Significant changes should be expected in metabolism and gene expression to adapt and fight against plant defense in larvae feeding in a resistant plant. Unfortunately, genome-wide data is not yet available on larvae feeding in resistant plants.

In this study, we have systematically analyzed changes in gene expression genome-wide through three-way comparisons: differences in gene expression between larvae feeding in a resistant plant versus those feeding in a susceptible plant at the same time; differences in gene expression between larvae feeding on resistant plants but at a different time; and differences in gene expression between larvae feeding in susceptible plants but at a different time. Our data revealed that overall 10%–18% of genes exhibited either greater or lesser transcript abundance in larvae feeding in resistant plants versus those feeding in susceptible plants. The proportions of genes with greater and lesser transcript abundance is relatively balanced except in 5-day larvae, which had more genes with greater transcript abundance in larvae from resistant plants. These differences were apparently caused by dynamic changes in gene expression in larvae due to feeding in either resistant or susceptible plants.

When the genes were classified into different functional categories and subcategories, more genes exhibited lesser transcript abundance in larvae from resistant plants, in the categories “nutrient metabolism”, “RNA metabolism”, and “protein metabolism” (Figure 2), particularly those genes involved in “tRNA synthesis”, “RNA transport”, “protein synthesis”, “protein folding”, and “citric acid cycle and energy metabolism” (Figure 3). Genes in the subcategories “tRNA synthesis”, “RNA transport”, “protein synthesis” and “protein folding” participate in protein synthesis either directly or indirectly. Genes in the subcategory “citric acid cycle and energy metabolism” participate in the production of energy and intermediates. Lower level expression of genes involved in citric acid cycle and protein synthesis would result in lower levels of energy, metabolic intermediates, and proteins available for larval growth and development. The reduction in the available supply of energy, intermediates, and proteins probably explains why Hessian fly larvae fail to develop into second instars before dying in resistant plants.

In contrast to genes involved in nutrient metabolism and protein synthesis, the number of P450 genes with greater transcript abundance was less different from the number of P450 genes with lower transcript abundance between larvae from resistant versus susceptible plants (Figure 4A). In fact, more P450 genes had greater transcript abundance than those with reduced transcript abundance in 0.5- and 5-day larvae from resistant plants, and more P450 genes were induced when larvae developed from 1- to 3-days and from 3- to 5-day again in resistant wheat (Figure 4B). Phylogenetic analysis revealed that the P450 genes with reduced transcript abundance in larvae from resistant plants are mainly distributed in two clusters, whereas the P450 genes with greater abundance are scattered in different clusters (Figure S2). We speculate that the P450s with less transcript abundance in larvae from resistant plants participate in internal physiological development processes, whereas the P450s with greater transcript abundance are likely to play important roles in counteracting host defense. P450s neutralize toxic chemicals which are either produced endogenously or ingested orally and perform a wide range of other physiological functions [24,25,26,27,28]. Our postulation is consistent with the fact that many P450 genes are expressed either exclusively or predominantly in Hessian fly larval midgut [34]. Greater expression levels of P450 genes were also found in soybean aphids feeding on resistant soybean plants [31]. In addition to P450s, a gene encoding a functionally-related enzyme, flavin-containing monooxygenase (dimethylaniline monooxygenase, Mdes007374), showed consistently high transcript abundance from larvae feeding in resistant plants (Table S2). Flavin-dependent monooxygenases are found as a detoxification mechanism in other insect species [35]. Similarly, several genes including Mdes009489 and Mdes007220, which encode protease inhibitors, exhibited transcript abundance in insects from resistant plants (Table S2). These inhibitors could suppress wheat proteases involved in immunity to avoid host defense reactions in response to herbivore attack [36]. Overall our data suggest that toxic defense play an important role in wheat resistance to Hessian fly larvae. We speculate that toxic chemicals slow down insect feeding, which gives resistant plants more time to strengthen cell walls and other host defenses, resulting in the eventual death of Hessian fly larvae due to lack of nutrients. Thus, the death of Hessian fly in resistant plants could be a combination of toxic defense and the strengthening of cell walls [19,20].

Young Hessian fly larvae produce and likely inject a large number of SSGPs into host plants during feeding [11,12,22,37]. One of the objectives of this study was to identify critical candidate effector proteins that are crucial for host manipulation. Based on our data, members in families 9, 8, and 16 are likely important for Hessian fly larvae to manipulate host plants. First, many members in these families exhibited greater transcript abundance in larvae from resistant plants (Figure 5). Since Hessian fly larvae feed inside wheat plants and cannot migrate, the only choice the larvae have in response to plant defense is to secrete more critical effectors into plants in order to survive. Therefore, those putative effector genes with greater levels of expression are likely critical for plant manipulation. Second, most members in these families of SSGPs are so-called early genes, namely they were expressed most abundantly in 0.5-day larvae, and expression levels then decreased as the larvae advanced into later stages (the right panel of Figure 5). Host plant manipulation is irreversible in the Hessian fly—wheat interaction [9]. We hypothesize that Hessian fly larvae inject early effectors into plants for host manipulation, and then stop producing these effectors when the larvae sense that plant manipulation has been accomplished. When larvae feed in resistant plants, they fail to manipulate plants, and therefore, continue to produce these effectors over a long time. In contrast to families 9, 8, and 16, genes in families 3 and 71 exhibited different expression patterns. Most members in these two families exhibited lower transcript abundance in 1- and 3-day larvae from resistant plants, and these genes belong to so called late genes, which were expressed most abundantly in 3-day larvae from susceptible plants. We speculate that these late SSGPs are injected into host plants for other functions, and one of the possible functions for the late effectors is to protect the attacked host plants from secondary infestation or infection. Susceptible plants become physically weak after Hessian fly attack, and therefore are vulnerable to microbes in the surrounding environment. Hessian fly larvae are parasites and when plants die, insects in the plants also die. SSGP gene families are large in Hessian fly, with >500 members in a family. Different family members are highly diversified and therefore could perform entirely different functions among family members. Members within the same family have exhibited different expression patterns. Therefore, further research with individual genes in different families is still needed to confirm their roles in host plant manipulation.

## 4. Materials and Methods

### 4.1. Insect

Hessian fly biotype GP was used in this study. The population was derived from a colony collected in Scott County, Kansas, in 2005 [38]. The colony has been maintained on the wheat cultivar “Karl 92” in the greenhouse since then.

#### 4.1.1. Wheat Cultivars, Infestation, and Sample Collection

Two isogenic wheat lines, “Newton” and “Molly”, were used as the host plants for Hessian fly infestation and sample collection. Hessian fly biotype GP was used in this study. Newton is a winter wheat line susceptible to Hessian fly, whereas Molly is a Hessian fly-resistant line (containing the resistance gene *H13*) derived from Newton through seven cycles of backcrossing [39]. For infestation and sample collection, 20 germinated wheat seeds were planted in 10-cm-diameter pots filled with PRO-MIX “BX” potting mix (Hummert Inc., Earth City, MO, USA) in a growth chamber programmed at 20:18 °C (Light:Dark) with a photoperiod of 14:10 (L:D) h. When wheat seedlings reached the 1.5 leaf stage (stage 11 on Zadoks scales), the plants were infested with an average 0.5 Hessian fly females per plant by confining the adult flies in a screened cage. After five days, eggs hatched into neonates that migrated into wheat plants. When the first larva was found at the feeding site, the time was set at zero and larval age started from that time. Larvae were collected at day 0.5, 1, 3, and 5, by dissecting plants to expose the insects. Since larvae become inactive in resistant plants, no samples were compared beyond this stage. The dissected plants were soaked in water in a micro-centrifuge. After enough insects were collected in a tube, the water was removed and larvae frozen in liquid nitrogen for RNA extraction. Three independent replicates were carried out for each time point.

#### 4.1.2. RNA Extraction and Quantification

Total RNA was extracted using TRI reagent (Molecular Research Center Inc., Cincinnati, OH, USA), according to the manufacturer procedures. RNA concentrations were determined using a Nanodrop *ND-2000* spectrophotometer (NanoDrop Technologies Inc., Wilmington, DE, USA). Quality of the RNA samples was further confirmed by analyzing the integrity of the samples on an Agilent TapeStation Bioanalzer (Agilent Technologies, Palo Alto, CA, USA). 

#### 4.1.3. RNA Library Construction and Sequencing

mRNA was selected through a oligo-dT column. Libraries were constructed from purified mRNA samples following the Illumina’s sample preparation instructions (Illumina, San Diego, CA, USA). Briefly, ~20 µg of total RNA from each sample was digested with DNase I (Sigma, St. Louis, MO, USA) to remove potential DNA contamination. mRNA was then purified by oligo(dT) magnetic beads and fragmented into 100–400 bp fragments. cDNA was produced from the RNA fragments using reverse transcriptase (Invitrogen, Carlsbad, CA, USA) with random hexamers as primers. An Agilent TapeStation Bioanalzer (Agilent Technologies, Palo Alto, CA, USA) was used to qualify and quantify the libraries. Libraries were sequenced using an Illumina HiSeq2000 system (Illumina Inc.).

#### 4.1.4. Analysis of RNA-Seq Data

Illumina sequence reads were processed to remove adaptors using Trimmomatic (version 0.32) [23] and the resulting reads were aligned to the Hessian fly draft genome sequence (https://i5k.nal.usda.gov/) [22] using Genomic Short-read Nucleotide Alignment Program (GSNAP) [40]. Uniquely aligned reads were used to determine the read depth per annotated gene in each sample by an in-house Perl script. To test the null hypothesis that no difference in gene expression existed of each gene between two groups, the generalized linear model method, assuming negative binomial distribution of read counts implemented in the DESeq2 package (version 1.4.5), was used to compute a *p*-value for each gene [41]. The parameters of “Independentfiltering = yes” in DESeq2 were used to filter genes that were unlikely to be differentially expressed. The genes left from the filtering were kept as informative genes. An FDR (false discovery rate) approach was adapted to convert *p*-values to *q*-values to account for multiple tests [42]. Genes with *q*-values no larger than 5% were declared to be differentially expressed.

### 4.2. BLASTX Search to Annotate Transcripts

Sequences of a set of Hessian fly transcripts (*n* = 18,832) were searched against the GenBank non-redundant protein squence database (nr) using BLASTX to identify homologous hits. For each transcript, only the best hit with the *E*-value no larger than 1 × 10^−30^ was reported.

#### 4.2.1. Classification of Genes According to Their Functions

Based on the Genbank search results, the genes with known functions were divided into eight functional categories based on their GO terms (http://www.uniprot.org/uniprot/) [43]. The eight categories are “nutrient metabolism”, “reduction/oxidation (redox) and detoxification”, “structure and adhesion”, “RNA metabolism”, “protein metabolism”, “transport”, “regulatory proteins” and “SSGPs”. Each category was further divided into sub-categories, again based on their GO terms. The subcategories were described in the results section and in reference [21].

#### 4.2.2. qRT-PCR Validation of Transcript Abundance

To validate changes observed in RNA-seq analyses, primers covering 12 representative transcripts selected from different categories were designed using the tool available on the website http://www.ncbi.nlm.nih.gov/tools/primer-blast/ (Figure S3). The primer sets were used for qRT-PCR analysis. DNase-treated RNA was used as template for cDNA synthesis using random hexamers with an iScript cDNA synthesis kit (BioRad, Hercules, CA, USA), according to the manufacturer’s guidelines. Samples were then treated with RNase H (Invitrogen). cDNA was quantified on a Nanodrop ND-2000 spectrophotometer (NanoDrop Technologies Inc.) and samples were diluted to 15 ng/µL to ensure equal amounts of cDNA template for quantification of mRNA abundance.

qRT-PCR was conducted on an Applied Biosystems StepOne plus machine with SYBR Green I (Applied Biosystems, Foster City, CA, USA). The following parameters were used: 95 °C for 10 min, 40 cycles of 95 °C for 3 s and 60 °C for 30 s. Transcript abundance of the gene encoding a hexokinase (Mdes009091) was used as an endogenous control in qRT-PCR. This gene is expressed relatively equally in all stages according to RNA-seq data. Quantification of transcript levels detected by qRT-PCR was based on the Relative Standard Curve Method). Statistical significance for the log-transformed arbitrary expression values was analyzed by ANOVA using the PROCMIXED procedure of SAS (SAS institute Inc., SAS/STAT User’s Guide, Version 9.13). Data from three biological replicates (each replicate assayed two times in independent qRT-PCR experiments) were combined and included as a random effect in the analysis. Log fold-change calculations were performed by comparing transcript abundance of the selected genes in larvae feeding in resistant plants with transcript abundance for the same genes in larvae feeding in susceptible plants. Fold-change was considered statistically significant if the *p*-value was <0.05.

## Supplementary Materials

Supplementary materials can be found at www.mdpi.com/1422-0067/17/8/1324/s1.
