# Supplementary material for: Genes Expressed Differentially in Hessian Fly Larvae Feeding in Resistant and Susceptible Plants
Source: Int J Mol Sci. 2016 Aug 12;17(8):1324. doi: 10.3390/ijms17081324 (PMC5000721; doi:10.3390/ijms17081324)
Supplement: Supplementary file 1 [file ijms-17-01324-s001.zip › ijms-138969-Supplementary Materials/ijms-138969-supplmentary information.pdf]

Supplementary Materials: Genes Expressed Differentially in Hessian Fly Larvae Feeding in Resistant and Susceptible Plants

Ming-Shun Chen, Sanzhen Liu, Haiyan Wang, Xiaoyan Cheng, Mustapha El Bouhssini and R. Jeff Whitworth

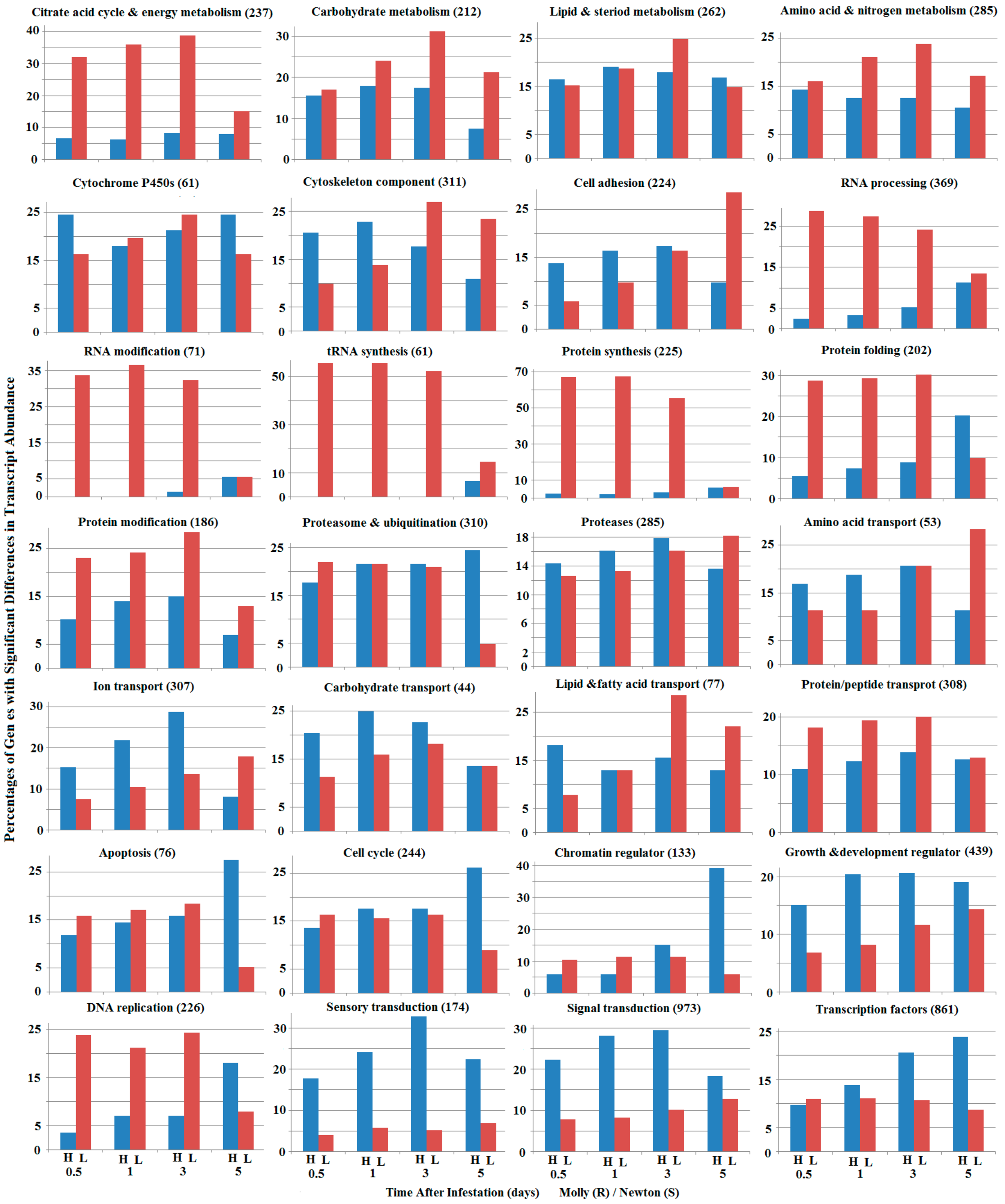

Figure S1. Percentages of genes in different subcategories with higher (blue bars) and lower (red bars) transcript abundance between larvae feeding in resistant (Molly) versus susceptible (Newton) plants at 0.5, 1, 3, and 5 days. Gene subcategories are marked on the top of each graph. The numbers of genes in each subcategory are given in parenthesis.

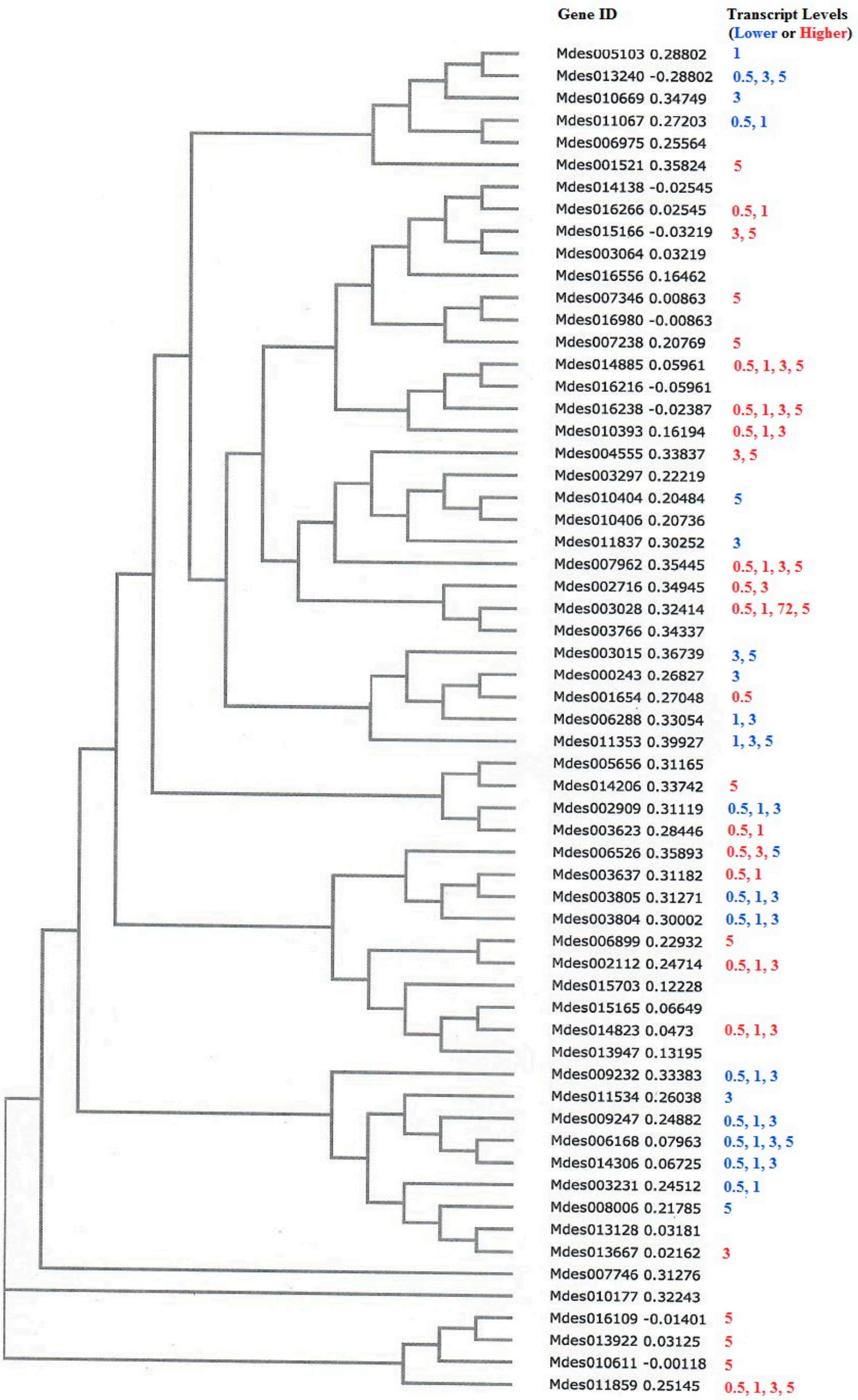

Figure S2. Cluster of putative P450s from Hessian fly genome. The cluster was generated using Clustal omega (http://www.ebi.ac.uk/Tools/msa/clustalo/) with putative protein sequences. The numbers 0.5, 1, 3, and 5 on the right indicate days the larvae fed in plants, and the gene with red or blue number(s) indicates significant differences in transcript abundance between larvae feeding in resistant plants versus those feeding in susceptible plants at that specific time. A red number indicates higher transcript abundance whereas a green number indicates lower transcript abundance in larvae feeding in resistant plants compared with those feeding in susceptible plants at that specific time.

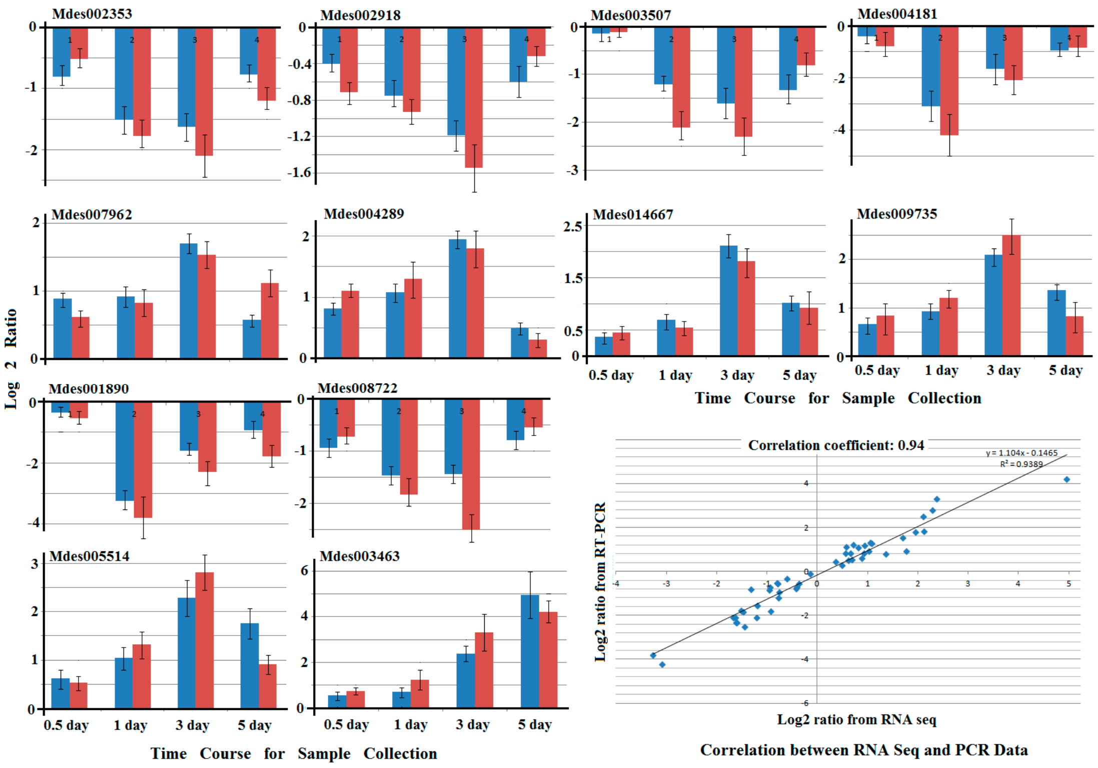

Figure S3. Validation of RNA seq data by Real-Time PCR**.** Blue bars represent Log2 fold change from RNA seq data and red bars represent Log2 fold change from PCR data. The last graph shows the correlation between these two data sets. Gene ID is on top of each graph. Three replicates were conducted for each treatment. Standard error is shown on each graph.

Table S1. Functional categories and subcategories of informative gene models (genes). Functional categories are highlighted with green whereas subcategories are highlighted with yellow.

Table S2. Pairwise comparisons between samples from larvae feeding in resistant plants and those from susceptible plants at the same time. Functional categories are highlighted with green whereas subcategories are highlighted with yellow. The table is divided into eight sub-tables. Sub-Table A: Genes with higher transcript abundance in larvae feeding in resistant plants for 0.5 day compared to the corresponding samples from larvae feeding in susceptible plants. Sub-Table B: Genes with lower transcript abundance in larvae feeding in resistant plants for 0.5 day compared to the corresponding samples from larvae feeding in susceptible plants. Sub-Table C: Genes with higher transcript abundance in larvae feeding in resistant plants for 1 day compared to the corresponding samples from larvae feeding in susceptible plants. Sub-Table D: Genes with lower transcript abundance in larvae feeding in resistant plants for 1 day compared to the corresponding samples from larvae feeding in susceptible plants. Sub-Table E: Genes with higher transcript abundance in larvae feeding in resistant plants for 3 days compared to the corresponding samples from larvae feeding in susceptible plants. Sub-Table F: Genes with lower transcript abundance in larvae feeding in resistant plants for 3 days compared to the corresponding samples from larvae feeding in susceptible plants. Sub-Table H: Genes with higher transcript abundance in larvae feeding in resistant plants for 5 days compared to the corresponding samples from larvae feeding in susceptible plants. Sub-Table I: Genes with lower transcript abundance in larvae feeding in resistant plants for 5 days compared to the corresponding samples from larvae feeding in susceptible plants.

Table S3. Pairwise comparisons between samples from larvae feeding in the same type of host plants (either resistant or susceptible), but at different times. Functional categories are highlighted with green whereas subcategories are highlighted with yellow. The table is divided into twelve sub-tables. Sub-Table A: Genes with higher transcript abundance in larvae feeding in resistant plants for 1 day compared to larvae for 0.5 day. Sub-Table B: Genes with lower transcript abundance in larvae feeding in resistant plants for 1 day compared to larvae for 0.5 day. Sub-Table C: Genes with higher transcript abundance in larvae feeding in resistant plants for 3 days compared to larvae for 1 day. Sub-Table D: Genes with lower transcript abundance in larvae feeding in resistant plants for 3 days compared to larvae for 1 day. Sub-Table E: Genes with higher transcript abundance in larvae feeding in resistant plants for 5 days compared to larvae for 3 days. Sub-Table F: Genes with lower transcript abundance in larvae feeding in resistant plants for 5 days compared to larvae for 3 days. Sub-Table G: Genes with higher transcript abundance in larvae feeding in susceptible plants for 1 day compared to larvae for 0.5 day. Sub-Table I: Genes with lower transcript abundance in larvae feeding in susceptible plants for 1 day compared to larvae for 0.5 day. Sub-Table J: Genes with higher transcript abundance in larvae feeding in susceptible plants for 3 day compared to larvae for 1 day. Sub-Table K: Genes with lower transcript abundance in larvae feeding in susceptible plants for 3 days compared to larvae for 1 day. Sub-Table L: Genes with higher transcript abundance in larvae feeding in susceptible plants for 5 days compared to larvae for 3 days. Sub-Table M: Genes with lower transcript abundance in larvae feeding in susceptible plants for 5 days compared to larvae for 3 days.
